# A Global Health Partnership's Use of Time-Limited Support to Catalyze Health Practice Change: The Case of GAVI's Injection Safety Support

**DOI:** 10.1371/journal.pone.0012986

**Published:** 2010-09-27

**Authors:** Ann Levin, Arnold Fang, Peter M. Hansen, David Pyle, Ousmane Dia, Nina Schwalbe

**Affiliations:** 1 Bethesda, Maryland, United States of America; 2 GAVI Alliance, Geneva, Switzerland; 3 John Snow International, Arlington, Virginia, United States of America; The George Washington University Medical Center, United States of America

## Abstract

This paper presents the findings of a study to assess the effectiveness and sustainability of a GAVI (Global Alliance of Vaccines and Immunization) sponsored, time-limited Injection Safety (INS) support. The support came in two forms: 1) in-kind, in the form of AD syringes and safety boxes, and 2) in cash, for those countries that already had a secure, multi-year source of AD syringes and safety boxes, but proposed to use INS support to strengthen their injection safety activities. In total, GAVI gave INS support for a three-year period to 58 countries: 46 with commodities and 12 with cash support. To identify variables that might be associated with financial sustainability, frequencies and cross-tabulations were run against various programmatic and socio-economic variables in the 58 countries. All but two of the 46 commodity-recipient countries were able to replace and sustain the use of AD syringes and safety boxes after the end of their GAVI INS support despite the fact that standard disposable syringes are less costly than ADs (10–15 percent differential). In addition, all 12 cash-recipient countries continued to use AD syringes and safety boxes in their immunization programs in the years following GAVI INS assistance. At the same time, countries were often not prepared for the increased waste management requirements associated with the use of the syringes, suggesting the importance of anticipating challenges with the introduction of new technologies. The sustained use of AD syringes in countries receiving injection safety support from GAVI, in a majority of cases through government financing, following the completion of three years of time-limited support, represents an early indication of how GHPs can contribute to improved health outcomes in immunization safety in the world's poorest countries in a sustainable way.

## Introduction

Global health partnerships (GHPs) have assumed an increasingly important role in financing global health [1], [2] and influencing related policies and activities at the country and global levels [3]. Yet the base of empirical evidence on the sustainability of GHP support remains limited. In at least 16 countries, GHP commitment is so large that it exceeds 5% of a government's expenditure [1], leading to concerns that low-income countries may not be able to match the ongoing costs once the funding ends, and that the support may pose major challenges in terms of macroeconomic management.

This paper uses evidence from the GAVI Alliance's (formerly Global Alliance for Vaccines and Immunization) Injection Safety Support program to assess the effectiveness and sustainability of time limited support in changing country practices known to lead to improved immunization safety. The GAVI Alliance, formed in 1999, serves to promote and finance new and under-utilized vaccines, as well as to improve immunization services. It is considered a global health partnership since it maintains a collaborative relationship among multiple organizations that share risks and benefits in pursuit of a shared goal [4].

Starting in 2002, GAVI offered Injection Safety Support to countries with per capita income of less than $1000 as one of its funding windows. The recent evaluation of this program is a prime opportunity for understanding the sustainability issues associated with time-limited GHP funding to low-income countries.

The GAVI Injection Safety Support window was meant to be catalytic and time-limited. In line with GAVI funding policies, countries with a Gross National Income (GNI) per capita below US$1,000 in 2003 qualified for this support. Countries approved for support would receive stocks of injection safety supplies to cover National Immunization Program (NIP) injections for children and women. For countries that already had funding for injection safety supplies, they could instead apply for cash support for program-strengthening activities related to injection safety. The objective of the program was to catalyze recipient countries to introduce auto-disable (AD) syringes and continue to use these following the conclusion of the three-year time-limited support from GAVI, with government financing or joint government and donor financing. For countries already using injection safety supplies (twelve countries), the objective was to support their continued use.

The supplies funded in the Injection Safety Support window are AD syringes and safety boxes for disposal of the used syringes. AD were developed by WHO in 1986 to counter the health threats posed by a) inappropriate handling and use of syringes designed to be reusable through proper sterilization and b) inappropriate reuse of standard, disposable syringes. The AD syringe is a single-use device and has a mechanism that disables the plunger after its first use. This support, which began in 2002, was complementary to GAVI's provision of new and underused vaccines. Since all new and underused vaccines offered by GAVI were provided jointly with injection safety supplies, Injection Safety Support only provides AD syringes and safety boxes for other NIP program vaccines (e.g. BCG, measles, DTP). By the end of 2006, 58 countries had completed their three years of injection safety support at a total cost of $110 million.

The adoption of AD syringes is cost-effective [5] from a societal perspective – i.e. the additional cost of using AD syringes is relatively small while the benefits of avoiding needle re-use are very pronounced. GAVI's injection safety support could potentially catalyze safe injection practices, and reduce blood-borne diseases such as Hepatitis B and C and Human Immunodeficiency Virus (HIV). At the time when GAVI was started, unsafe injections were estimated to be accountable for 1.3 million early deaths each year, and up to 40% of all injections in the world were given with reused syringes without sterilization [6].

This study investigates whether countries will continue using a new injection technology after a time-limited support of a GHP ends despite the fact that the standard injection supplies are less costly (10–15 percent differential). The factors that affected a country's likelihood of continuing the use of the new technology are identified. In addition, the program's impact on the use of infection safety supplies in the broader health sector was also examined.

## Methods

The study sample includes the 58 countries (see Table 1 for list of countries and Table 2 for selected characteristics) that completed their three years of Injection Safety Support by the end of 2006. The key outcome of interest is whether all 58 countries were able to sustain the use of AD syringes and safety boxes after GAVI support had ended. For this study, sustainability is considered to include two aspects, namely, *replacement* and financial sustainability. *Replacement* of GAVI support is considered the minimum requirement for injection safety practices to be sustained. *Replacement* is considered to have occurred when a country continues the use of AD syringes and safety boxes after GAVI INS support has ended, irrespective of the source(s) of financing. *Financial sustainability* takes into account the concept of self–sufficiency and is defined as the extent to which GAVI INS support is sustained through the resources of the country itself rather than external partners. The four levels of financial sustainability were the following: (1) None: AD syringes not replaced or partially replaced; (2) Low: replaced but fully donor dependent; (3) Medium: replaced with mixed government and donor funding; and (4) High: replaced with full domestic funding.

**Table 1:** List of Countries Receiving GAVI Injection Safety Support.

2002–2004	2003–2005	2004–2006
Armenia	Azerbaijan	Afghanistan (cash)
Burundi	Bhutan	Albania
Cambodia	Burkina Faso	Angola
Djibouti (cash)	Cameroon	Bangladesh
Ethiopia	CAR	Bolivia
Gambia	Comoros	Chad
Georgia	Congo DR	China (cash)
Korea DR	Ghana (cash)	Congo
Lao PDR	Indonesia	Eritrea
Senegal	Kenya	Guinea
Sierra Leone	Lesotho	Haiti (cash)
Sudan	Mali	Honduras (cash)
Uganda	Mozambique	Kyrgyz Rep.
Yemen (cash)	Myanmar	Mauritania (cash)
Zambia	Nepal	Niger
	Pakistan	Sri Lanka
	Rwanda (cash)	Tajikistan
	Sao Tome	Turkmenistan
	Somalia (cash)	Ukraine
	Tanzania (cash)	Vietnam (cash)
	Togo	Zimbabwe
	Uzbekistan	

**Table 2:** Characteristics of the 46 commodity-recipient countries and 12 cash-recipient countries.

		Number (%)	
Characteristics	Categories	Recipients of supplies	Recipients of cash
Year support ended	2004	**13 (28%)**	**2(17%)**
	2005	**18(39%)**	**4(33%)**
	2006	**15(33%)**	**6(50%)**
Regions (based on WHO categorization)	Africa	25 (54%)	4 (33%)
	Latin America	1(2%)	2 (17%)
	Eastern Mediterranean	2 (4%)	0(0%)
	Europe	9(20%)	4(33%)
	South-east Asia	7(15%)	0(0%)
	West Pacific	2(4%)	2(17%)
GNI per capita	< = $350	13(28%)	2(17%)
	$350–700	14(30%)	3(25%)
	>$700	17(37%)	5(42%)
	No data	2(4%)	2(17%)

The data collection involved three methods: 1) Review of available documentation on injection safety and waste management in the 58 countries including country applications for funding, annual progress reports, government and partner meetings, and WHO/UNICEF statistics on country immunization coverage, injection safety, program financing, and healthcare waste management; 2) telephone interviews with NIP or Ministry of Health program managers; and 3) telephone interviews with WHO and UNICEF regional and country focal points. To verify the findings, country reports on the use of injection safety supplies after GAVI funding ended were checked against data from UNICEF Supply Division on the procurement of AD syringes and safety boxes. By mid-2008 when the data was collected, each country had been without GAVI support for at least two years.

**Figure 1:**
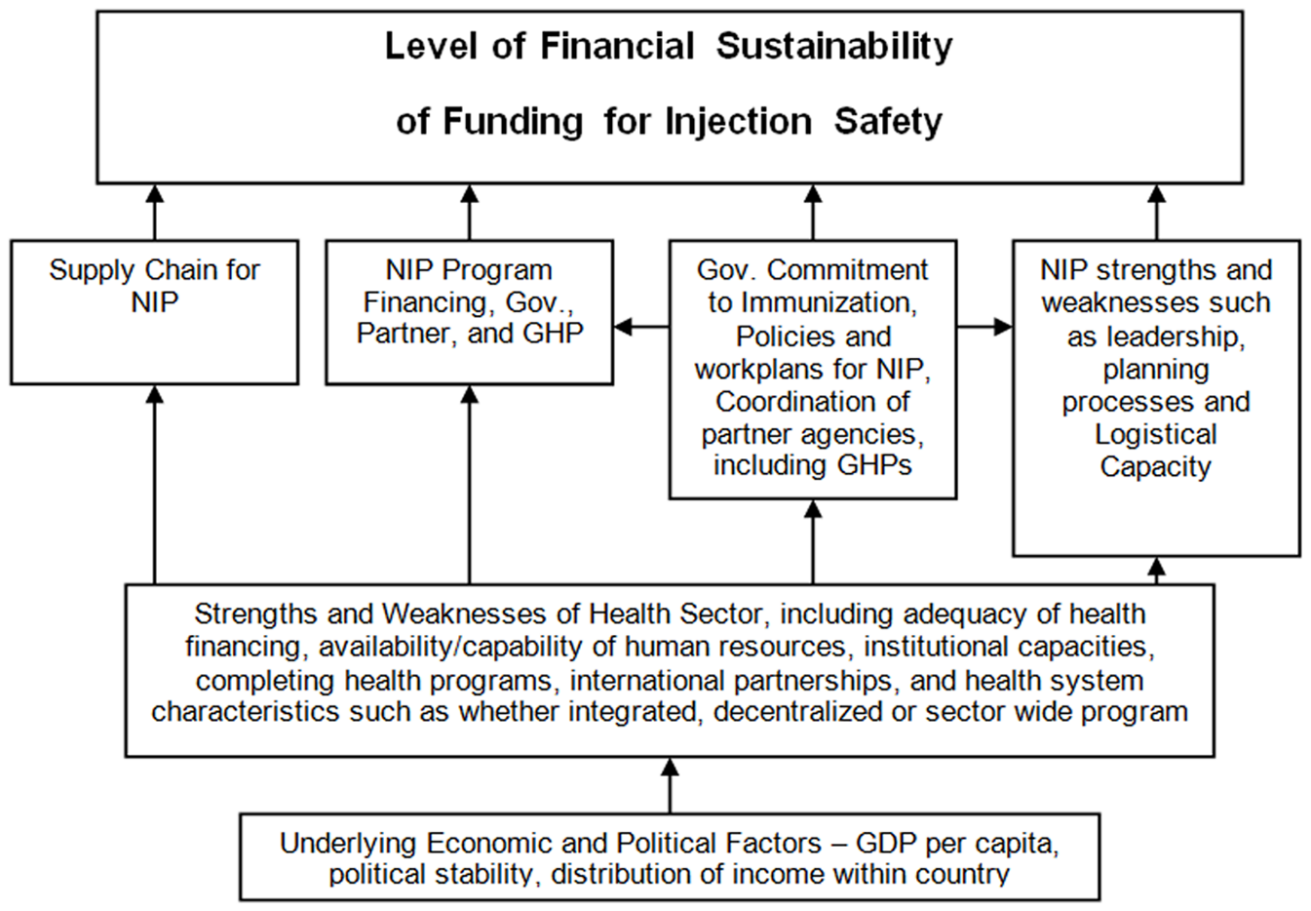
Financial Sustainability Framework. This figure describes the levels of the health system that affect financial sustainability of funding for injection safety. The abbreviations in the article are the following: NIP  =  National Immunization Program, GDP  =  Gross Domestic Production, and GHP  =  Global Health Partnership.

In the analysis, the study team evaluated the importance of factors that affect the extent to which AD syringes and safety boxes were funded with government funding, using the financial sustainability framework in Figure 1. The variables created for the analysis of the sustainability of injection safety support are shown in Table 3.

**Table 3:** Variables on Sustainability of Injection Safety Support.

OUTCOME VARIABLE	
Score/Range	**Level of sustainability of injection safety Support in Immunization Program**
1	Discontinued use of AD syringes and safety boxes
2	Dependent on donors to finance
3	Government is financing a proportion of the expenditures
4	Government is financing all AD syringes and safety boxes

DEPENDENT VARIABLES	
	**GNI per capita**
1–3	Score based on whether country has a per capita income ≤$350, $351–700, or >$700
	**Region**
1–6	Six regions are Western Pacific, Southeast Asia, Africa, Latin America, Europe and Eastern Mediterranean
	**Program strength**
1–5	Score based on whether DTP3 coverage ≥80 percent, country has a national injection safety policy, NIP manager rated the immunization program logistics as mostly or completely adequate, injection safety has a strong advocate in the MOH or partner organization and program maintains a continuous supply of AD syringes and safety boxes
	**Year that support ended**
1–3	The years that support ended range from 2004 to 2006.
	**Decision-making**
1–3	Score based on whether the adequacy of decision-making process on replacement was rated as none or little, some or excellent

To identify variables that might be associated with financial sustainability, frequencies and cross-tabulations are run against various programmatic and socio-economic variables in the 58 countries. The analysis is stratified by whether the country had introduced a new injection technology, i.e. received commodities, or received funding to supplement its already existing injection safety activities, i.e. received cash.

A key limitation of the study lay in its retrospective nature and its reliance on NIP managers. In some countries, NIP staff turnover meant that the managers were not at their posts when the application was written, or when the decision was made to replace GAVI's Injection Safety Support. Also, some NIP managers were not very knowledgeable about injection safety practices in the broader health sector. A further limitation was the fact that GAVI's injection safety support had only ended in 2006 in 21 out of 58 countries.

## Results

### Financial sustainability among the 46 Commodity-recipient Countries

In terms of sustainability, all but two of the 46 commodity-recipient countries (ninety-six percent) continued to use AD syringes and safety boxes after GAVI support ended. By mid-2008, more than half (25 countries, fifty-four percent) of these countries were financing the purchase of their own AD syringes and safety boxes completely with government funding and seven (15 percent) were using a mixture of government and donor funding. Only 12 (26 percent) countries continued to be totally donor dependent (see Table 4 for a summary).

**Table 4:** Financial sustainability among commodity and cash recipients.

Level of financial sustainability	Number (%) of Countries	
	Recipients of supplies	Recipients of cash
None: Total/partial discontinuation of AD syringes	2 (4%)	0 (0%)
Low: Fully donor-dependent for financing	12 (26%)	4 (33%)
Medium: Mixture of government and donor funding	7 (15%)	3 (25%)
High: Fully government funded	25 (54%)	5 (42%)
**Total**	**46 (100%)**	**12 (100%)**

Two of the 58 countries in the study at least partially discontinued the use of AD syringes for different reasons. Ukraine discontinued its use of AD syringes and safety boxes and opted instead to use its local production of standard disposable syringes. The second country, Uzbekistan, discontinued the use of AD syringes and safety boxes in three-quarters of its health facilities due to its decentralized procurement system. That is, since local governments in Uzbekistan have decentralized procurement and are responsible for expenditure related to primary care units [7], some authorities at the oblast level chose non-AD products rather than AD products.

### Determinants of financial sustainability among the 46 Commodity-recipient Countries (Bivariate results)

**Figure 2:**
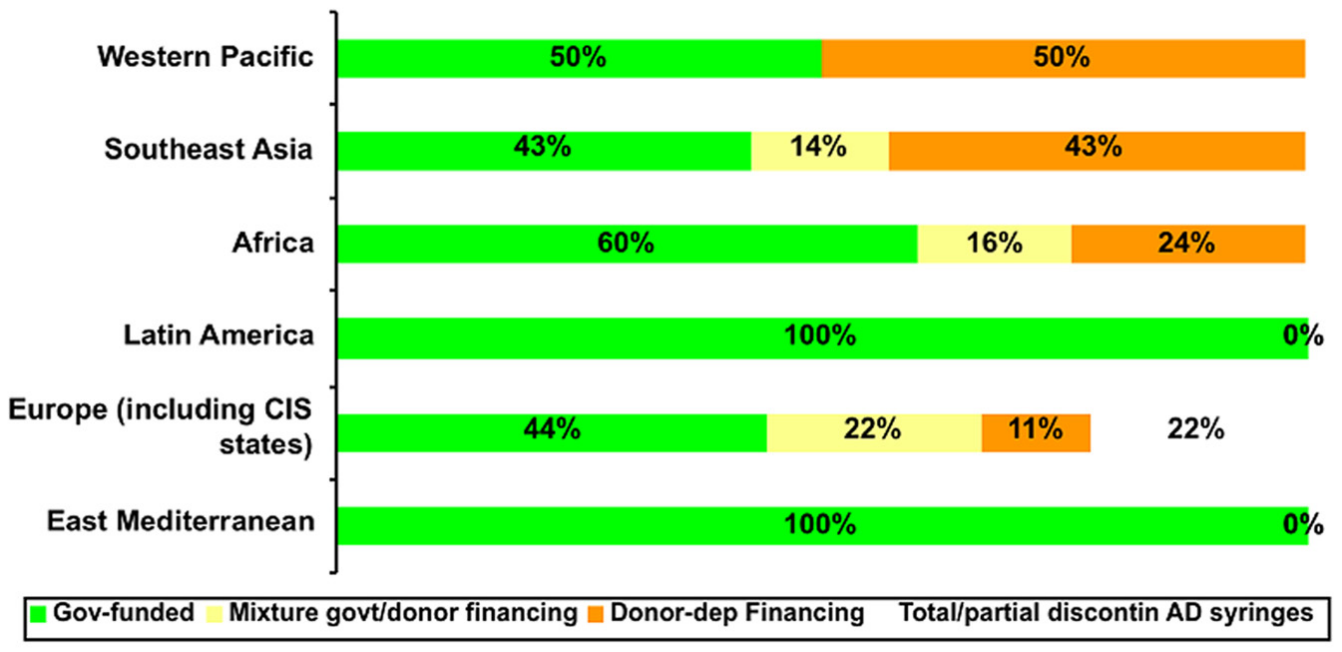
Financial sustainability by region. This figure shows the extent to which countries in different regions are using government funding to purchase AD syringes after GAVI funding ended. The green section of each bar represents the percent of countries that are fully government funded, the yellow section represents the countries that use a mixture of government and donor financing, the orange section is for countries that only use donor financing, and the clear section is for countries that have totally or partially discontinued the use of AD syringes.

When grouped by WHO region, the percentage of countries that funded their AD syringes and safety boxes from a government source ranged from 43 percent in the South-east Asia (three countries) to 100 percent in Latin America (three countries) and Eastern Mediterranean (two countries) (see Figure 2).

**Figure 3:**
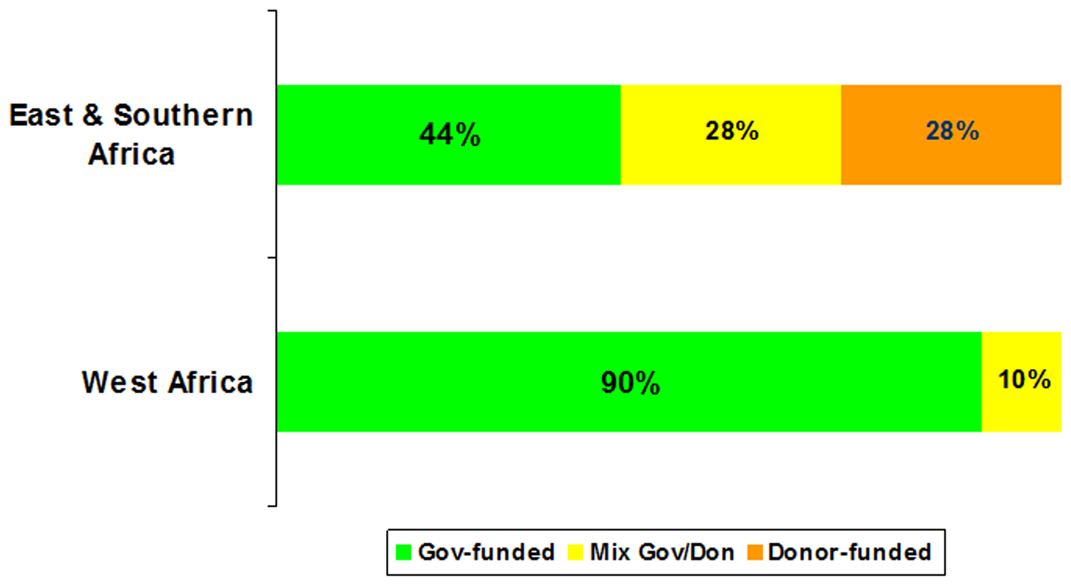
Financial sustainability in Africa. This figure shows the extent to which countries in Africa are using government funding to purchase AD syringes after GAVI funding ended. The green section of each bar represents the percent of countries that are fully government funded, the yellow section represents the countries that use a mixture of government and donor financing, and the orange section of the light is for countries that only use donor financing.

Some sixty percent of African countries had government funding for their AD syringes and safety boxes. However, this percentage was higher in West Africa (90%) than in other parts of Africa (see Figure 3). The explanation for this difference may be attributed to effective advocacy by WHO and UNICEF technical focal persons and donor budget support for the national immunization programs including vaccines and essential supplies. In addition, the European Union (EU) requested donor-assisted countries in West Africa to establish MOH budget line items for vaccines and injection safety commodities when they received support. Part of the Vaccine Independence Initiative (VII), the EU helped countries to purchase vaccines and injection safety commodities by guaranteeing the payment.

Seventy one percent of countries with a per capita income over USD 700 were fully government funded for AD syringes and safety boxes after the conclusion of GAVI's support, compared to 54% or less for the countries in the poorer income groups, but this factor was not statistically significant (see Table 5).

**Table 5:** Financial sustainability of Countries by Per Capita Income Level.

GNI per capita (U.S.$)	≤$350 (%)	$351–$700 (%)	>$700 (%)	Total
*None:* Total/Partial discontinuation of AD syringes	0 (0)	1 (7)	1 (6)	2 (5)
*Low:* Fully Donor-dependent for Financing	5 (38)	3 (24)	2 (12)	10 (23)
*Medium:* Mixture of Government and Donor funding	1 (8)	4 (29)	2 (12)	7(18)
*High:* Fully Government Funded	7 (54)	6 (43)	12 (71)	25(54)
**Total**	**13**	**14**	**17**	**44**

**Pearson Chi Square  = 7.774 (p<0.456).**

Countries with stronger programs were slightly more likely to be able to replace GAVI funding with government funding, or with a mixture of government and donor funding than were countries with medium or weak program strength (Table 6). (See explanation of the program strength variable in Table 3.) It is, however, clear that this factor alone does not account for financial sustainability because three countries with weak programs have full government funding for AD syringes and safety boxes and two countries with high program strength have total or partial discontinuation of AD syringes.

**Table 6:** Financial sustainability of Countries by program strength.

Level of Sustainability	Weak (program strength 1–2) (%)	Medium (program strength 3) (%)	High (program strength 4–5) (%)	Total Number (%)
*None:* Total or partial discontinuation of AD syringes	0 (0)	0 (0)	2 (9)	2 (5)
*Low:* Fully Donor-dependent for Financing	2 (40)	5 (45)	2 (9)	9 (23)
*Medium:* Mixture of Government and Donor funding	0 (0)	1 (10)	6(26)	7 (18)
*High:* Fully Government Funded	3 (60)	5 (45)	13 (57)	21 (54)
**Total**	**5**	**11**	**23**	**39**

Pearson chi-square  = 15.747 (p<0.471).

Countries that ended their support in 2004 were more likely (62 percent) by the time of the evaluation to have secured government funding for their AD syringes and safety boxes than countries ending in 2005 (56 percent) or 2006 (47 percent) (Table 7). Countries with a better decision-making process, based on whether program managers rated the adequacy of the process on replacement as excellent, some, little or none, also were more likely to have government funding for AD syringes and safety boxes. (Table 8).

**Table 7:** Financial sustainability of Countries by year in which GAVI Injection Safety Support ended.

Level of financial sustainability	2004	2005	2006	Total (%)
*None:* Total or partial discontinuation of AD syringes	0 (0%)	1 (6%)	1 (7%)	2 (4%)
*Low:* Fully donor dependent for financing	4 (31%)	5 (28%)	3 (20%)	12 (26%)
*Medium:* Mixture of government and donor funding	1 (8%)	2 (11%)	4 (27%)	7 (15%)
*High:* Fully government funded	8 (62%)	10 (56%)	7 (47%)	25 (54%)
**Total**	**13**	**18**	**15**	**46**

Pearson chi-squared  = 5.829 (p<0.666).

**Table 8:** Financial Sustainability of Countries by Adequacy of Decision-making.

	Little or No Process	Some	Excellent	
*None:* Total or partial discontinuation of AD syringes	1 (25)	1 (10)	0 (0)	2
*Low:* Fully Donor-dependent for Financing	1 (25)	0 (0)	7 (28)	8
*Medium:* Mixture of Government and Donor funding	2 (50)	2 (20%)	3 (12)	7
*High:* Fully Government Funded	0 (0)	7 (70%)	15 (45)	22
**Total**	4	10	25	39

Pearson Chi-square  = 36.752 (p<0.013).

### Financial Sustainability of Countries receiving Cash instead of Injection Safety Materials

All 12 cash recipient countries continued to use AD syringes and safety boxes in their NIP after the end of GAVI support. Among them, five purchased AD syringes and safety boxes with full government funding. Among the other seven countries, three purchased supplies using a combination of government and donor support, while the remaining four countries continued to use only donor funding (refer to Table 4 for a summary).

Cash-recipient countries mainly used GAVI funding to support injection safety in their programs. Four of the 12 countries (Afghanistan, Djibouti, Haiti and Honduras) used the support to reinforce the use of AD syringes and safety boxes through program areas such as training, monitoring, supervision and evaluation. Three countries (Ghana, Mauritania and Tanzania) used the funding to construct incinerators for disposal of used syringes. Two countries (China and Vietnam) used GAVI INS support to purchase AD syringes and safety boxes from local manufacturers per their agreements with GAVI. In the three countries (Rwanda, Somalia and Yemen) that used the funding to purchase AD syringes and safety boxes internationally, GAVI made agreements to transfer cash to these countries because of their particular circumstances. In Somalia, for example, an agreement was made with UNICEF to purchase the syringes so that it could continue to provide commodities to a country which is considered “fragile.” Another country – Yemen, was already using its World Bank loan to purchase AD syringes, and used GAVI INS cash support to replace this source of funding.

### Impact on broader health sector

The majority of program managers interviewed stated that GAVI's injection safety support was influential on the decision to introduce AD syringes and safety boxes into other health services. Thirty countries, (51% of total countries), introduced AD syringes and safety boxes partially (20 countries) or fully (10 countries) into other non-immunization services. In 21 of the countries, AD syringes were introduced into other services during or after the GAVI support took place while 9 countries already were using these products.

Program managers also stated that GAVI's support influenced the development of injection safety policies for the health sector. Of the 39 countries that had a policy or were developing one, 35 program managers stated that GAVI's support influenced the development of this policy by increasing their awareness of injection safety issues.

The introduction of AD syringes also resulted in additional waste disposal requirements in many of the countries. Program managers in a third of the countries (19) stated that health care waste management is an unresolved problem for their health systems. While the study confirmed that 42 out of the 58 countries had a policy on health care waste management, about one-third (19) of program managers considered it an unresolved problem. Sixteen reported a lack of incinerators, one cited difficulties maintaining incinerators, and two mentioned unsafe disposal of waste.

### Comparison of use of AD syringes in GAVI INS recipients with non-GAVI lower-middle income countries

The use of AD syringes is higher in GAVI INS-supported countries than in non-GAVI lower middle-income countries (Table 9). However, it is not only the lack of eligibility for GAVI support that is affecting these countries. Discussions with regional WHO/UNICEF focal points indicate that some middle-income countries in the EUR and AMR regions do not perceive that AD syringes are necessary for their countries because they do not believe that their health facilities re-use single-use syringes. However data from this evaluation indicated that health ministry officials wanted to continue using AD syringes once they had been introduced into their country's immunization programs.

**Table 9:** Type of syringes used in GAVI INS countries and non-GAVI lower-middle income countries.

Type of syringes used in immunization program	GAVI INS recipients	Non-GAVI lower-middle income countries
Fully using AD syringes	56 (96)	11 (41)
Using mixture of AD and non-AD syringes	1 (2)	10 (37)
Fully using non-AD syringes	1 (2)	6 (22)

Source: JRF 2007 and telephone interviews with program managers and WHO and UNICEF staff.

## Discussion

GAVI's injection safety support was instrumental in accelerating the introduction of AD syringes and safety boxes in low-income countries. Although the support was limited to three years, this evaluation study showed that almost all countries receiving the support (96%) continued to procure and utilize AD syringes and safety boxes in the years after GAVI support came to an end. The countries continued use of the AD syringes despite the fact that they were more costly than standard disposable syringes (10–15 percent differential). Furthermore, a majority of the countries receiving support continued the use of these devices using only government funding. In addition, the use of AD syringes is higher in GAVI INS-supported countries than in non-GAVI lower middle-income countries. These findings demonstrate some evidence of the potential impact that GHPs can achieve through targeted time-limited support to countries.

One of the reasons for GAVI's success was that AD syringes were only slightly more costly than standard disposable syringes at the time of evaluation. From 1992 to 2001, the average price of an AD syringe decreased from U.S. $0.13 to U.S. $0.06 (UNICEF Supply Division). GAVI launched its support after this substantial drop in price, and thus did not drive any further significant reductions in price. By the time GAVI countries were expected to assume the cost of procuring AD syringes, the price was sufficiently low to make it easier and more affordable for governments to begin procuring on their own. It is possible that if the price differential between the new and older technologies had been higher, that countries would be less likely to continue with the new technology.

Another factor in sustaining the use of injection safety supplies was the advocacy efforts of global and regional partners. These included WHO, UNICEF and the European Union. In the African region, for example, the regional WHO Task Force on Immunization (TFI) issued a statement in 2002 recommending the use of AD syringes for national immunization programs. The statement was used successfully to achieve a transition to this technology in most countries in this part of the region. Another partner, the European Union (EU), requested donor-assisted countries in West Africa to establish MOH budget line items for vaccines and injection safety commodities when they received support. This led to a higher level of sustainability observed in West Africa as compared to other parts of the continent.

The additional waste management requirements imposed by AD syringes were perceived by NIP managers interviewed and some regional WHO or UNICEF focal persons as a growing problem that needed addressing. Even though some countries received technical assistance and donor support to construct incinerators through non-GAVI initiatives (such as through disease-specific campaigns), they often lacked the scale or durability that was necessary for prolonged use. Coordinated, scaled-up efforts from donors and global health partnerships are therefore necessary for the implementation of health care waste management plans developed in the countries.

Our study showed that the exclusive use of AD syringes in immunization programmes in low-income countries could potentially be sustained in the long-term, which was contrary from what some critics wrote [8], [9] on the sustainability of this approach of achieving injection safety.

WHO estimates that only 3% of all injections are administered for immunization—95% of all injections occur in delivery of curative services [10]. The successful achievement of injection safety thus also lies in low-income countries' ability to reduce the number of inappropriate injections given. The dissemination of information, education and communication (IEC) materials and behaviour change campaigns targeting patients and health workers were thus identified by the Safe Injection Global Network (SIGN) as a key strategy in achieving injection safety.

Some spillover effects of introducing AD syringes in NIP towards the curative sectors were identified. However, a more comprehensive study will need to be done to tease out the intervention's impact and investigate whether other confounding effects on the timing of the introduction were taking place as well.

GAVI was successful in taking an innovative approach to enhancing injection safety among NIP in low-income countries. Through providing a catalytic, time-limited support in cash or supplies, countries were either able to replace unsafe injection safety supplies with AD syringes and safety boxes, or to enhance their existing injection safety initiatives through training, monitoring, local procurement or waste management of injection wastes. It suggests that low resource countries, which are recipients of time-limited in-kind or monetary support, can be induced or catalyzed to change their health practices.

### Conclusion

The sustained use of AD syringes in countries receiving injection safety support from GAVI, in a majority of cases through government financing, following the completion of three years of time-limited support represents an early indication of how GHPs can contribute to improved immunization safety in the world's poorest countries in a sustainable way. The support also had further-reaching effects in catalyzing the use of injection safety supplies in other services beyond NIP, and in influencing the development of a health-sector wide policy on injection safety.

The sustainability of GHP efforts hinges upon the ability of various actors to work in effective partnerships. The participation of implementing governments, donors and multilateral agencies is important, as shown in the decision-making and planning process of replacing GAVI's supply. It is critical that a plan be made for replacing donor funding in advance of the end of the time-limited support window. Regional variations show that the advocacy efforts of international agencies such as WHO and UNICEF are important in encouraging governments of these countries to take up the funding of injection safety themselves.

Although this study alone cannot provide a comprehensive conclusion on the sustainability of time-limited programs, it contributes important evidence on an issue of increasing importance. GHPs should design support mechanisms to increase the likelihood that governments will be able to sustain desired outcomes following completion of the time-limited support. Additional research studies are needed to better understand program characteristics that are associated with increased sustainability of GHP support. Rigorous monitoring and evaluation of programs is also needed, and should include ongoing monitoring following the completion of time-limited support to assess the degree to which desired outcomes are sustained over time.
